# No obvious advantage of hyperthermic intraperitoneal chemotherapy after interval debulking surgery in the treatment of advanced ovarian cancer: A retrospective study

**DOI:** 10.3389/fsurg.2022.997344

**Published:** 2022-09-12

**Authors:** Mengmeng Lyu, Jin Lu, Yang Shen, Qianqian Chen, Fei Deng, Jinhua Wang

**Affiliations:** Department of Gynecologic Oncology, Jiangsu Cancer Hospital / Jiangsu Institute of Cancer Research / The Affiliated Cancer Hospital of Nanjing Medical University, Nanjing, China

**Keywords:** HIPEC, ovarian cancer, interval debulking, neoadjuvant chemotherapy, retrospective

## Abstract

**Objective:**

To study the efficacy of interval debulking surgery (IDS) plus hyperthermic intraperitoneal chemotherapy (HIPEC) compared to IDS alone for the treatment of ovarian cancer after neoadjuvant chemotherapy (NACT).

**Methods:**

We conducted a retrospective study of patients with stage IIIC/IV high-grade serous ovarian carcinoma who underwent surgery at our center from January 2018 to December 2019. Patients who underwent IDS after NACT with (*N* = 20) or without (*N* = 42) HIPEC were included. HIPEC was administered after surgery and was combined with 1–2 courses of intraperitoneal hyperthermic perfusion with normal saline only. We analyzed clinical information and outcomes for the two groups.

**Results:**

The median progression-free survival (PFS) was 14.05 months in the IDS plus HIPEC group and 12.97 months in the IDS group (*P* = 0.597). The median overall survival (OS) was not reached. After adjustment for age between the two groups, the differences in PFS and OS remained nonsignificant. The change ratio of postoperative CA-125 to preoperative CA-125 was 0.66 in the IDS plus HIPEC group and 0.53 in the IDS group (*P* = 0.341). The difference in human epididymis protein 4 (HE-4) change ratio between the two groups was nonsignificant (*P* = 0.225). No significant difference was observed in the occurrence of grade 3 and 4 adverse events between the two groups (*P* = 0.201).

**Conclusion:**

After NACT, IDS plus HIPEC did not show significant PFS and tumor index change ratio benefits over IDS alone in patients with primary ovarian cancer. Further investigations are needed to assess the role of HIPEC in the treatment of ovarian cancer.

## Introduction

Based on incidence and mortality, ovarian cancer is among the top ten cancer types in females (1). A large proportion of ovarian cancers are at an advanced stage when discovered (i.e., FIGO stage III or IV), and the estimated 5-year survival rate is 20%–40% (2). According to the histological classification, epithelial cancer accounts for 90% of ovarian cancer types. The most common histologic subtype is high-grade serous ovarian cancer, accounting for more than 70% of epithelial ovarian cancers (2, 3). Patients with advanced ovarian cancer are typically treated with the standard treatment of cytoreductive surgery and adjuvant platinum-based chemotherapy or NACT (4). However, high recurrence and mortality remain unsolved problems in the treatment of ovarian cancer.

The concept of HIPEC was introduced early in the 1980s. High temperature alone can selectively kill tumor cells *via* multiple mechanisms. The combination of high temperature and chemotherapy can also function *via* multiple mechanisms, including increased local drug concentrations, cytotoxic effects of chemotherapeutic drugs on cancer cells, and penetration of the drug into tumor tissue (5, 6). HIPEC was first extensively used in gastroenteric peritoneal metastases, as supported by multiple clinical studies (7). Use of HIPEC in ovarian cancer began in the 1990s; however, the effect of HIPEC on ovarian cancer remains heavily debated (8).

Prospective randomized clinical evidence concerning HIPEC in ovarian cancer is limited (9). Two randomized clinical trials have demonstrated the efficacy of HIPEC in primary treated ovarian cancer after NACT and recurrent ovarian cancer (10, 11). However, these studies had limitations and were not convincing (9, 12). Other randomized trials (8, 13) and retrospective analyses (14) led to inconsistent conclusions and demonstrated a lack of efficacy with HIPEC in ovarian cancer.

HIPEC started to be used for ovarian cancer in 2018 in our center and was suspended in 2020 due to the coronavirus disease 2019 pandemic. Although this technology is not widely used in our center, we hope to share our limited clinical experience and data. In this article, we describe our retrospective study on the efficacy of HIPEC after NACT and IDS in patients with primary treated stage IIIC/IV high-grade serous ovarian cancer.

## Patients and methods

In this study, we selected data from patients who met the following conditions: diagnosed with stage IIIC/IV ovarian carcinoma with a histologic subtype of high-grade serous ovarian cancer at our center, underwent surgery from January 2018 to December 2019, underwent IDS after partial response to NACT with or without HIPEC, and had a postoperative residual tumor size of zero (R0) or diameter for residual tumor ≤1 cm (R1) (15). HIPEC was not a routine procedure after ovarian cancer surgery in our center and was administered based on the patient's voluntary choice. Patients who underwent IDS alone were selected from the same treating physicians as those who underwent HIPEC. And patients with postoperative residual tumor size >1 cm, or who didn't receive standard systemic chemotherapy after surgery were excluded from the study. Ethics approval was obtained from the ethics committee of The Affiliated Cancer Hospital of Nanjing Medical University (approval number 2022 ke-kuai 012). Informed consent was obtained from all patients.

The NACT and adjuvant chemotherapy regimen for all patients was platinum- and taxane-based, which is the classic first-line regimen for ovarian cancer. The number of cycles was 6–8 for most patients unless new progression occurred.

HIPEC using chemotherapy drugs was administered after surgery and was combined with 1–2 courses of intraperitoneal hyperthermic perfusion with normal saline only. Different perfusion courses were conducted on separate days. The intraperitoneal chemotherapy drugs used for HIPEC in our center include cisplatin, lobaplatin, and paclitaxel, either alone or in combination. Intraperitoneal perfusion tubes including two inflow and two outflow catheters were inserted into the abdominal cavity during the operation, and HIPEC with chemotherapy was administered within one week after the operation. Chemotherapy drugs were mixed with normal saline and administered intraperitoneally at a temperature of 43 °C for one hour. The additional intraperitoneal hyperthermic perfusion with only normal saline was also administered at a temperature of 43 °C for one hour. Intravenous chemotherapy drugs were administered intravenously on the same day as HIPEC.

For all patients, CA-125 and HE-4 serum levels were regularly tested before surgery and are referred to as preoperative CA-125 and preoperative HE-4, respectively. The tumor index was tested three weeks after the first cycle of postoperative chemotherapy, either intravenous or HIPEC, and before the second course of postoperative chemotherapy and is referred to as postoperative CA-125 and postoperative HE-4.

Postoperative complications were defined as complications that occurred within one month after the operation and were graded according to the Common Terminology Criteria for Adverse Events Version 5.0.

PFS was defined as the time between surgery and the first progression of the disease. Progression was defined as the date of imaging or biochemical progression occurrence according to RECIST 1.1 criteria and CA-125 criteria (16). OS was evaluated from IDS to the date of death. The change rate of CA-125 and HE-4 was calculated by postoperative CA-125 divided by preoperative CA-125 and by postoperative HE-4 divided by preoperative HE-4, respectively. The survival data were calculated using the Kaplan–Meier method, and the survival distribution was compared using the log-rank test. The results were adjusted for unbalanced characteristics between the two groups by a multivariate Cox proportional hazards regression model. Continuous variables were compared using Student's t test. Categorical variables were analyzed using the chi-squared test. A *P* value <0.05 was considered significant. All statistical calculations were performed using SPSS software (version 23.0).

## Results

From January 2018 to December 2019, there were 20 patients with stage IIIC/IV ovarian carcinoma with a histologic subtype of high-grade serous ovarian cancer and satisfactory cytoreduction surgery (R0 or R1) after NACT at our center who underwent HIPEC. They were included in the IDS plus HIPEC group. There were 42 patients who underwent IDS alone, were treated by the same physicians during the same period as the IDS plus HIPEC group, and were included in the IDS group.

The clinical characteristics of the study population are shown in Table 1. The average age of the patients was 52.75 years in the IDS plus HIPEC group and 57.64 years in the IDS group. Women in the IDS plus HIPEC group were younger than those in the IDS group (estimated mean difference of 4.89 years, *P* = 0.036). The majority of the patients (approximately 90%) in both groups were diagnosed with FIGO IIIC ovarian cancer, and the remaining patients had stage IV disease. The majority of the patients received ≤3 cycles of adjuvant chemotherapy.

**Table 1:** Patient characteristics.

Variable	IDS + HIPEC	IDS	*P* value^a^
No. of patients	20	42	
Age (years)(mean, range)^b^	52.75 (35–70)	57.64 (35–69)	0.036
FIGO stage- no. (%)			
IIIC	18 (90%)	37 (88%)	0.825
IV	2 (10%)	5 (12%)	
Tumor index
Preoperative CA-125, mean, U/mlb	197.76 (5.63–1228.9)	313.04 (10.73–2097)	0.343
Preoperative HE-4, mean, pmol/lb	146.14 (54.73–407.2)	198.97 (55.7–855.2)	0.387
Mean no. of cycles of NACT-no. (%)			
≤3	16 (80%)	36 (85.7%)	0.567
>3	4 (20%)	6 (14.3%)	

Abbreviations: HIPEC, hyperthermic intraperitoneal chemotherapy; IDS, interval debulking surgery; FIGO, International Federation of Gynecology and Obstetrics; NACT, neoadjuvant chemotherapy.

^a^ Student's t test was used to compare groups in age and tumor index, the Chi square test was used to compare groups in FIGO stage, mean cycles of NACT.

^b^ Data in parentheses represent minimum and maximum of values for each group.

For women who underwent IDS plus HIPEC, HIPEC with chemotherapeutic drugs was mostly administered within one week after surgery. Intravenous chemotherapy was administered primarily on the same day as HIPEC. Eleven patients underwent HIPEC with cisplatin at a dose of approximately 75 mg/m^2^ combined with intravenous paclitaxel (approximately 175 mg/m^2^). Three patients underwent HIPEC with paclitaxel (approximately 175 mg/m^2^) combined with intravenous carboplatin (AUC5-6). One patient underwent HIPEC with lobaplatin (approximately 30 mg/m^2^) combined with intravenous paclitaxel (approximately 175 mg/m^2^). Four patients underwent HIPEC with cisplatin (75 mg/m^2^) and low-dose paclitaxel (approximately 60 mg/m^2^) combined with intravenous paclitaxel chemotherapy (approximately 175 mg/m^2^). One patient underwent HIPEC with lobaplatin (approximately 30 mg/m^2^) and low-dose paclitaxel (approximately 60 mg/m^2^) combined with intravenous paclitaxel (approximately 135 mg/m^2^). HIPEC with two different chemotherapy drugs was performed on two separate days.

The median follow-up time after surgery was 30.35 months. Of the 20 patients in the IDS plus HIPEC group, 12 had experienced disease progression at the follow-up cutoff, and the median PFS was 14.05 months (95% CI [3.45; 24.66]). Of the 42 patients in the IDS group, 32 had experienced disease progression at the follow-up cutoff, and the median PFS was 12.97 months (95% CI [7.39; 18.54]). There was no significant difference between the two groups (*P* = 0.597). At the cutoff, 5 of the 20 patients in the IDS plus HIPEC group and 12 of the 42 patients in the IDS group had died. The median OS was not reached.

**Figure 1:**
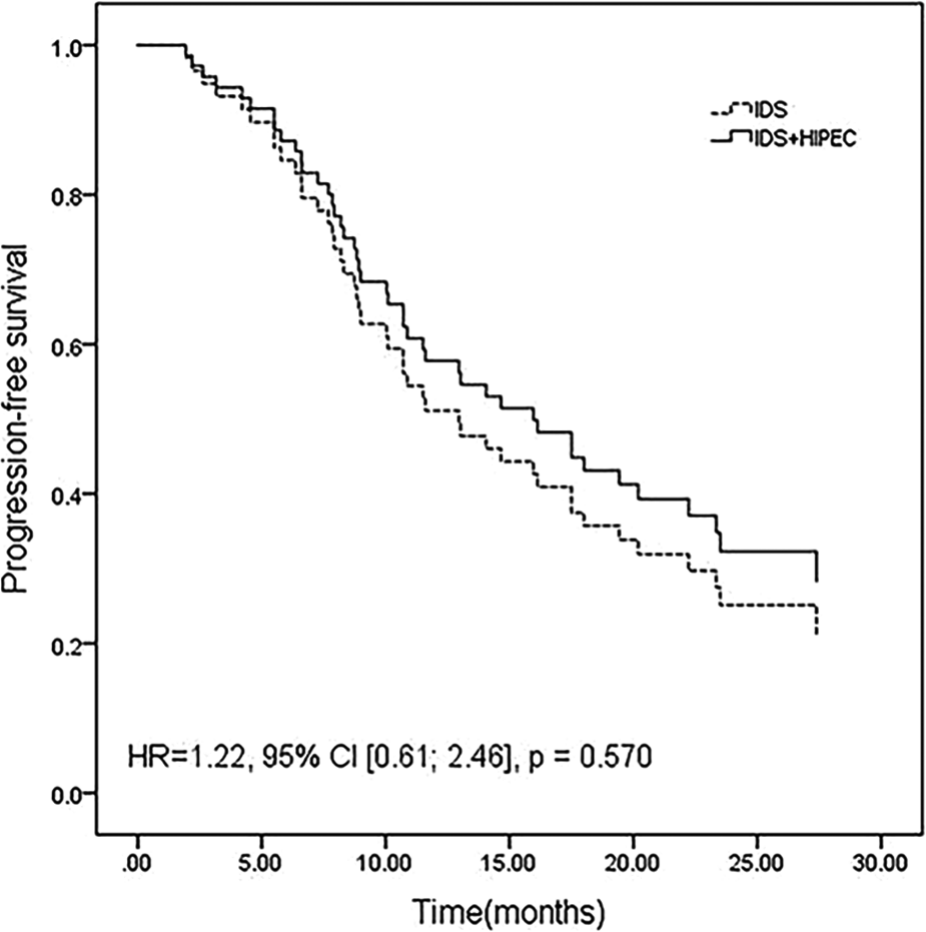
Progression-free survival adjusted for age by a multivariate Cox proportional hazards regression model.

**Figure 2:**
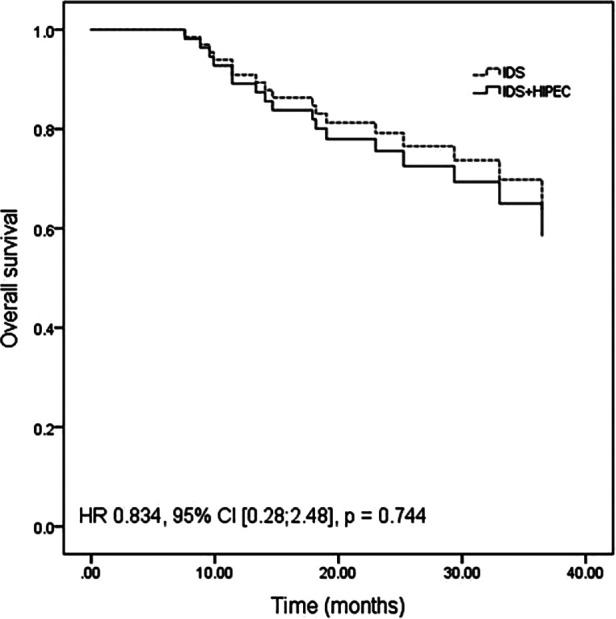
Overall survival adjusted for age by a multivariate Cox proportional hazards regression model.

After adjustment for age between the two groups, the differences in PFS (HR 1.22, 95% CI [0.61; 2.46], *P* = 0.570) (Figure 1) and OS (HR 0.834, 95% CI [0.28; 2.48], *P* = 0.744) (Figure 2) remained nonsignificant.

**Figure 3:**
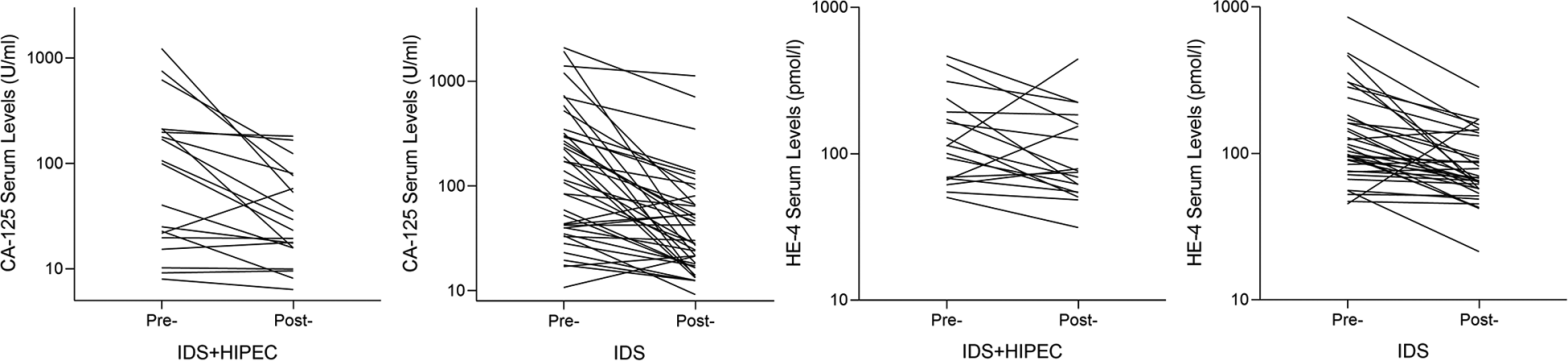
Change curve of preoperative and postoperative CA-125 and HE-4 serum levels. Pre, Preoperative; Post, Postoperative; HIPEC, hyperthermic intraperitoneal chemotherapy; IDS, interval debulking surgery.

The average preoperative CA-125 level was 197.76 U/ml in the IDS plus HIPEC group and 313.04 U/ml in the IDS group. The average postoperative CA-125 level was 47.53 U/ml in the IDS plus HIPEC group and 94.70 U/ml in the IDS group, which showed no significant difference (*P* = 0.319). The average change ratio of postoperative CA-125 to preoperative CA-125 was 0.66 in the IDS plus HIPEC group and 0.53 in the IDS group, which also showed no significant difference (*P* = 0.341) (Table 2) (Figure 3).

**Table 2:** CA-125 and HE-4 Serum Levels.

	IDS + HIPEC	IDS	*P* value^d^
CA-125 (U/ml)			
Preoperative^a^	197.76	313.04	0.343
Postoperative^b^	47.53	94.70	0.319
Change ratio (post/pre)^c^	0.66	0.53	0.341
HE-4 (pmol/l)			
Preoperative^a^	146.14	198.97	0.387
Postoperative^b^	118.84	100.23	0.499
Change ratio (post/pre)^c^	0.95	0.71	0.225

Abbreviations: HIPEC, hyperthermic intraperitoneal chemotherapy; IDS, interval debulking surgery

^a^ CA-125 and HE-4 tested before surgery.

^b^ CA-125 and HE-4 tested after the first cycle of postoperative chemotherapy either intravenously or HIPEC, and before the second course of postoperative chemotherapy.

^c^ “postoperative CA-125” divided by “preoperative CA-125”, “postoperative HE-4” divided by “preoperative HE-4”.

^d^ Student's t test was used to compare the two groups.

The average preoperative HE-4 was 146.14 pmol/L in the IDS plus HIPEC group and 198.97 pmol/L in the IDS group. There was no significant difference in the preoperative HE-4 between the two groups (*P* = 0.387). There was no significant difference in the postoperative HE-4 and HE-4 change ratios between the two groups (*P* = 0.499 and *P* = 0.225, respectively) (Table 2) (Figure 3).

There were no postoperative deaths in either group. The grade 3 and 4 adverse events were mainly surgical complications and chemical drug toxicities. The most common side effects were hematological, including intra-abdominal hemorrhage and toxicities caused by chemotherapy. In the IDS plus HIPEC group, two patients experienced gastrointestinal adverse events; one patient experienced acute kidney injury that was associated with the use of cisplatin; and one patient had anastomotic leakage approximately 20 days after the operation. No significant difference was observed in the occurrence of grade 3 and 4 adverse events between the two groups (*P* = 0.201) (Table 3).

**Table 3:** Grade 3 and 4 complications.

	IDS + HIPEC (20)	IDS (42)	*P* ^b^
Grade 3 and 4 complications^a^	9 (45%)	12 (28.6%)	0.201
Hematological	5	11	
Gastrointestinal	2	0	
Abdominal infection	0	1	
Acute kidney injury	1	0	
Anastomotic leak	1	0	

Abbreviations: HIPEC, hyperthermic intraperitoneal chemotherapy; IDS, interval debulking surgery.

^a^ Complications occurred within one month after operation and were graded according to CTCAE Version 5.0.

^b^ Chi square test was used to compare the two groups.

## Discussion

As ovarian cancer typically spreads intraperitoneally, intraperitoneal chemotherapy has been proposed as a therapy for advanced ovarian cancer for years. Clinical trials, such as GOG172, a randomized, phase 3 trial, demonstrated that intraperitoneal chemotherapy improves survival in patients with optimally debulked stage III ovarian cancer compared with intravenous chemotherapy (17, 18). However, there are conflicting clinical data. A randomized phase 3 clinical trial with 1560 participants enrolled (GOG-252) revealed no significant increase in PFS with intraperitoneal chemotherapy compared with intravenous chemotherapy and that intraperitoneal chemotherapy was less tolerated when intraperitoneal cisplatin was used (19). Intraperitoneal chemotherapy is not recommended as a standard of care as a first-line treatment for ovarian cancer (20).

There are few randomized clinical trials on HIPEC in the primary treatment of ovarian cancer. Based on the results of trials, the role of HIPEC in ovarian cancer remains debatable. Van Driel et al. (10) conducted a multicenter, open-label, phase 3 trial that enrolled 245 patients to investigate the function of HIPEC in patients with stage III ovarian cancer who were undergoing NACT and interval cytoreductive surgery. Patients were randomly assigned after three cycles of NACT and interval cytoreductive surgery. The median recurrence-free survival was longer in the group with HIPEC than in the group without HIPEC (14.2 months vs. 10.7 months). The median OS was longer in the HIPEC group (45.7 months vs. 33.9 months). Furthermore, HIPEC was not found to increase the rates of side effects. However, this trial can be criticized for several aspects (12). In a randomized trial conducted by Lim et al. (8, 13), a survival analysis did not show the statistical superiority of HIPEC. In patients who received NACT, there was no significant difference in the median PFS for the HIPEC and control groups (20 months vs. 19 months, respectively). The median OS for the HIPEC and control groups was similar (54 months and 51 months, respectively).

Retrospective studies also reached different conclusions on the role of HIPEC in primary ovarian cancer. One retrospective study (21) conducted by Ziying Lei et al. showed that primary cytoreductive surgery with HIPEC was associated with better long-term survival. A retrospective study (22) conducted by Jieun Ko et al. demonstrated that consolidation HIPEC has no specific survival benefit for patients with advanced ovarian cancer after completion of first-line treatment. A study (23) conducted by Jessica Jou et al. resulted in an unexpected conclusion; the study revealed that HIPEC was associated with an increased risk for platinum-refractory or resistant disease in ovarian cancer patients who underwent NACT and IDS, while no benefit was shown in median PFS.

HIPEC is controversial not only in primary treated ovarian cancer but also in recurrent ovarian cancer. A prospective study (24) conducted by Spiliotis et al. resulted in a positive conclusion but was highly criticized due to methodological issues (8, 25). A meta-analysis of the use of HIPEC in recurrent ovarian cancer demonstrated positive results in the improvement of OS (26). In addition, the safety of HIPEC repetition in recurrent ovarian cancer treatment has also been studied, which has revealed encouraging data (27). HIPEC combined with minimally invasive surgery for platinum-sensitive single recurrent ovarian cancer has also been shown to be safe and effective (28). Other studies have revealed negative results (5). For example, a study conducted by Glauco Baiocchi et al. showed that the addition of HIPEC to cytoreduction surery did not improve survival in recurrent ovarian cancer (29). However, the role of HIPEC in primary and recurrent ovarian cancer needs further validation.

The results of our study showed a median PFS of 14.05 months in the IDS plus HIPEC group and 12.97 months in the IDS group (*P* = 0.597). There was no significant difference between the change ratio of postoperative CA-125 to preoperative CA-125 or the ratio of postoperative HE-4 to preoperative HE-4 between the two groups. Even after adjusting for the age differences between groups, the differences in PFS and OS continued to lack statistical significance (*P* = 0.570 and 0.744, respectively). In our study, neither the survival analysis nor the change in tumor index showed the advantage of HIPEC in patients who underwent IDS after NACT.

In our study, HIPEC was administered using chemotherapy drugs, with additional intraperitoneal hyperthermic perfusion using only normal saline. Intraperitoneal hyperthermic perfusion with normal saline has been proven to improve survival outcomes in pancreatic cancer without increasing the risk of complications (30, 31). Thus, the combination of HIPEC with chemotherapy drugs and normal saline was thought to strengthen the antitumor effect of HIPEC. However, our findings did not demonstrate a significant increase in PFS in patients who received HIPEC after IDS.

HIPEC is often administered immediately after cytoreduction surgery (5), but some institutions administer HIPEC on days after surgery rather than at the end of surgery (32). The intention of cytoreduction surgery for ovarian cancer is to remove visible lesions; thus, surgical excision is extensive, and the operation time is long (6). In addition, cytoreduction surgery sometimes includes gastrointestinal anastomosis. However, the safety of HIPEC after gastrointestinal anastomosis remains uncertain (5). In our institute, HIPEC with chemotherapy drugs is not routinely performed immediately after surgery and is usually performed within one week after IDS with a closed technique, as we hope to guarantee patient safety and increase patient tolerance. However, the delay in HIPEC at our center may affect the efficacy of HIPEC.

Chemotherapy drugs used in HIPEC in published studies differed and contained multiple types, including cisplatin, paclitaxel, and doxorubicin (26, 33). Cisplatin has been used more often and is recommended in the NCCN guidelines. Drugs used in our study included cisplatin, lobaplatin, and paclitaxel, either alone or in combination, which was not identical to previously reported usage (34). In fact, not only do the types of chemotherapy drugs used in HIPEC differ in reported articles but parameters including drug dosage, goal temperature, and duration of perfusion also vary widely (5). A consensus on these aspects is still lacking.

There are some limitations of our study. Most importantly, this is a retrospective study with a small sample size, which may make the results inaccurate. The time of HIPEC administration and the technique and drugs used for HIPEC in our study were not identical to those in previously reported studies. These differences may affect the clinical results.

To date, HIPEC remains a controversial treatment for ovarian cancer. Large clinical randomized trials are needed to address this topic and assess the true role and benefit of HIPEC in the treatment of ovarian cancer.

## Data Availability

The raw data supporting the conclusions of this article will be made available by the authors, without undue reservation.
